# Differential regulation of cell death pathways by the microenvironment correlates with chemoresistance and survival in leukaemia

**DOI:** 10.1371/journal.pone.0178606

**Published:** 2017-06-05

**Authors:** Malak Yahia Qattan, Emyr Yosef Bakker, Ramkumar Rajendran, Daphne Wei-Chen Chen, Vaskar Saha, Jizhong Liu, Leo Zeef, Jean-Marc Schwartz, Luciano Mutti, Constantinos Demonacos, Marija Krstic-Demonacos

**Affiliations:** 1 College of Applied Medical Sciences and Community Services (CAMS&CS), King Saud University, Riyadh, Saudi Arabia; 2 School of Environment and Life Sciences, University of Salford, Salford, United Kingdom; 3 School of Pharmacy, International Medical University, Kuala Lumpur, Malaysia; 4 Faculty of Biology, Medicine and Health, University of Manchester, Manchester, United Kingdom; 5 Tata Translational Cancer Research Centre, Kolkata, India; University of Pittsburgh, UNITED STATES

## Abstract

Glucocorticoids (GCs) and topoisomerase II inhibitors are used to treat acute lymphoblastic leukaemia (ALL) as they induce death in lymphoid cells through the glucocorticoid receptor (GR) and p53 respectively. Mechanisms underlying ALL cell death and the contribution of the bone marrow microenvironment to drug response/resistance remain unclear. The role of the microenvironment and the identification of chemoresistance determinants were studied by transcriptomic analysis in ALL cells treated with Dexamethasone (Dex), and Etoposide (Etop) grown in the presence or absence of bone marrow conditioned media (CM). The necroptotic (RIPK1) and the apoptotic (caspase-8/3) markers were downregulated by CM, whereas the inhibitory effects of chemotherapy on the autophagy marker Beclin-1 (BECN1) were reduced suggesting CM exerts cytoprotective effects. GCs upregulated the RIPK1 ubiquitinating factor BIRC3 (cIAP2), in GC-sensitive (CEM-C7-14) but not in resistant (CEM-C1-15) cells. In addition, CM selectively affected GR phosphorylation in a site and cell-specific manner. GR is recruited to RIPK1, BECN1 and BIRC3 promoters in the sensitive but not in the resistant cells with phosphorylated GR forms being generally less recruited in the presence of hormone. FACS analysis and caspase-8 assays demonstrated that CM promoted a pro-survival trend. High molecular weight proteins reacting with the RIPK1 antibody were modified upon incubation with the BIRC3 inhibitor AT406 in CEM-C7-14 cells suggesting that they represent ubiquitinated forms of RIPK1. Our data suggest that there is a correlation between microenvironment-induced ALL proliferation and altered response to chemotherapy.

## Introduction

Leukaemia is a cancer characterised by aberrant proliferation of white blood cells and may be acute/chronic and myeloid/lymphoblastic. Approximately 80% of childhood ALL patients reach remission [1]. Topoisomerase II inhibitors and GCs are used to treat ALL [2]. Drug toxicity and chemoresistance are major challenges and the outcome for patients who fail therapy remains poor, increasing the necessity for more potent, less toxic therapies. GCs are used to treat ALL [3–5] as they induce leukocyte cell death through the glucocorticoid receptor (GR) [6]. Upon entering the cytoplasm, GCs bind to GR causing dissociation from heat shock proteins, translocation into the nucleus and regulation of target genes [7, 8]. GCs utilise mainly the intrinsic apoptotic pathway [9–13] modulating the gene expression of the pro-apoptotic BCL-2-interacting mediator of cell death (Bim) [14], as well as fine tuning the balance between NOXA and Mcl-1 [10].

The synthetic glucocorticoid Dexamethasone (Dex) and the topoisomerase II inhibitor Etoposide (Etop) act via GR and p53 respectively. Etoposide-dependent cell death is partly mediated by the induction of Bax, Puma and NOXA through p53 activation [15]. Both p53 and GR affect other pathways that regulate cell fate such as autophagy or necroptosis, potentially through the regulation of the autophagy marker BECN1 [16, 17] or the key modulator of necroptosis RIPK1 (receptor interacting serine-threonine kinase 1) respectively [18].

GR function is controlled at multiple levels, including protein stability, cofactor interactions and post-translational modifications [10, 19–24]. GR phosphorylation modulates transcriptional activity and cellular response to GCs by altering cofactor recruitment, nuclear/cytoplasmic location, proteasomal degradation and protein half-life [10, 25, 26]. GR phosphorylation is differentially regulated in sensitive versus resistant ALL [10] and in particular ratio of GR phosphorylation at Ser211 versus Ser226 is higher in sensitive to GCs ALL cells. GR phosphorylation at Ser211 is mediated by cyclin-dependent kinases and p38-MAPK pathway, while Ser226 is targeted by c-Jun N-terminal kinases (JNK) [10, 23, 24, 27, 28]. Ser211 is hyperphosphorylated after hormone binding whereas phosphorylation of GR at Ser226 is associated with nuclear export, GR sumoylation and suppression of its transcriptional activity [20, 24, 27].

Drug resistance and cancer progression are mediated by several factors including communication between the bone marrow microenvironment and leukaemia cells in a two-way exchange of regulation [29, 30]. Different modes of communication are involved such as soluble factors and direct cell-cell contact [31–33]. Furthermore, inflammation, oxidative stress and different types of cell death have been implicated in determining leukaemic cell fate, depending on the drugs used and exposure to the microenvironment [10, 29, 34, 35]. However, better understanding of the role of the bone marrow microenvironment in leukaemia is important, given its impact on clinical outcomes.

In this study the effect of the microenvironment on ALL cells exposed to individual and combined treatments was investigated. Transcriptome analysis was performed and alterations in gene expression followed. Furthermore, the effects of the combinatory drug treatment and CM on GR phosphorylation status, GR phosphoisoforms transcriptional selectivity and cell fate were explored.

## Methods

### Cell lines and treatments

CEM-C1-15 (C1, GC-resistant cells) and CEM-C7-14 (C7, GC-sensitive cells), MOLT4 ALL and K562 chronic myeloid leukemia cell lines were cultured in Roswell Park Memorial Institute-1640 (RPMI-1640, Sigma-Aldrich) medium supplemented with Dextran Coated Charcoal treated serum (DCC) (Hyclone) used during experiments. Bone marrow cell-conditioned media (CM) was generated from the supernatant of HS5 cells after incubation with serum-free RPMI -1640 for 48 hours. Leukaemic cells were treated with CM at a final ratio of 1/6^th^ CM for 48 hours.

### Western blot analysis

Cells were seeded with DCC-FBS RPMI media in the presence or absence of CM, treated with Dex/Etop for the indicated times and western blot analysis performed as described previously [36]. Cells were lysed in high salt lysis buffer and protein concentration was determined using the BioRad assay. Antibodies recognizing actin (Sigma-Aldrich), GR (H300, Santa Cruz Biotechnology), GR phosphorylated at S211 (Abcam), GR phosphorylated at S226 (Abcam), BECN1 (Abcam), RIPK1 (Santa Cruz Biotechnology) and caspase-3 (New England Biolabs) were used. ImageJ was used for quantifying blots [37].

### Transcriptome analysis

Total mRNA was extracted from C7 cells treated with Dex, Etop and CM in varying combinations using the RNeasy Plus Mini Kit and QIAshredder spin columns according to manufacturer’s guidelines (Qiagen). The extracted RNA was supplied to the Genomic Technologies Core Facility, Manchester University. GeneChip Human Genome U133 plus 2.0 Array was used to analyse expression profiles in cells as described previously [38]. Background correction, quintile normalization, and gene expression analysis were performed using the robust multiarray average (RMA) in Bioconductor software [39]. Principal component analysis (PCA) was performed with Partek Genomics Solution (v6.5). Differential expression analysis was performed using the Limma package in the Bioconductor software [40]. Two-way comparisons were performed. Gene lists of differentially expressed genes were controlled for false discovery rate (FDR) errors using QVALUE [41].

Profile filtering was used to cluster genes based on expression profile similarity across the dataset [42]. A list of differentially-expressed genes was created by filtering for probesets with a q-value less than 0.05 and fold change greater than or equal to 2.0. Clustering was performed on the means of each sample group (log_2_) that had been z-transformed (for each probeset the mean set to zero, standard deviation to 1). K-means clustering was done on the basis of similarity of profiles (Manhattan Distance) across the dataset using the "Super Grouper" plug-in of maxdView software. This method clusters all genes that have the same trend of expression across different treatments. Data were segregated into eight clusters based on expression profile similarity. DAVID was used for functional gene annotation.

### Caspase-8 assay

Cells were incubated with CM, Dex and Etop for 48, 36 and 24 hours respectively, harvested and processed according to manufacturer’s guidelines [43]. In brief, 5μl diluted FLICA (fluorochrome-labeled inhibitor of caspases assay) reagent was added to 93μl cell suspension, supplemented with 2μl 500 μg/ml Hoechst 33342. Following 1 hour incubation at 37°C, two washes with Apoptosis Wash Buffer, cells were centrifuged and resuspended in 100μl of Apoptosis Wash Buffer supplemented with 10μg/ml propidium iodide and analysed using the NucleoCounter NC-3000.

### Quantitative RT-PCR

Quantitative RT-PCR analysis was performed as described previously [44]. Total RNA was extracted from the cells using RNeasy Plus Mini Kit (Qiagen, USA) and 1μg total RNA from each sample was converted to cDNA according to the BioScript reverse transcriptase (Bioline) two-step protocol using an oligo-dT primer (Bioline). cDNA was subjected to qPCR analysis using SensiFAST SYBR No-ROX Kit (Bioline). The primers used for Rpl19, RIPK1, BECN1 and BIRC3 are listed in Table A in S1 File.

### Chromatin immunoprecipitation (ChIP)

Chromatin immunoprecipitation assays were performed as described previously [45]. Proteins were crosslinked to DNA by 1% formaldehyde. Crosslinking was quenched by adding 125mM glycine. Cells were washed twice with PBS and once with ChIP Buffer 1 and ChIP Buffer 2, then resuspended in 3ml of ChIP Buffer 3. Chromatin was sheared to approximately 300bp via sonication (Diagenode Bioruptor) and subjected to immunoprecipitation using antibodies against total GR (Diagenode), Ser211-phosphorylated GR and Ser226-phosphorylated GR. Anti-rabbit IgG was used as a negative control. Precipitated DNA-protein complexes were reverse cross-linked by incubating with elution buffer (50mM Tris-HCl; pH 8, 100mM EDTA and 1% SDS w/v) at 65°C for 16 hours. DNA was purified using QIAquick PCR Purification Kit and subjected to qPCR using primers flanking the putative GREs in the BIRC3, BECN1 and RIPK1 promoters (Table B in S1 File). Data for each antibody was normalised to the respective negative control IgG.

## Results

### Transcriptome analysis

In order to establish the experimental conditions to analyse the effects of clinically used drugs and the microenvironment on ALL cells, effects of glucocorticoid Dex and Etop were analysed by assessing the viability of the resistant CEM-C1-15 and the sensitive CEM-C7-14 ALL cells to glucocorticoid and anthracycline induced apoptosis, as well as K562 chronic myelogenous leukemia (CML) cell lines, using trypan blue exclusion assay (Figs A and B in S1 File). It was observed that treatment with 1μM Dex for 36hrs and 10μM Etoposide for 24hrs displayed optimal effect on ALL GC sensitive cells, whereas CML cells were largely GC-resistant. Conditioned media obtained from bone marrow stromal cell line HS5 was used to mimic the effect of the microenvironmental soluble factors on ALL cells and its amount and duration of incubation chosen at 1/6^th^ CM/total media and 48 hours of treatment (Fig C in S1 File and data not shown).

**Fig 1:**
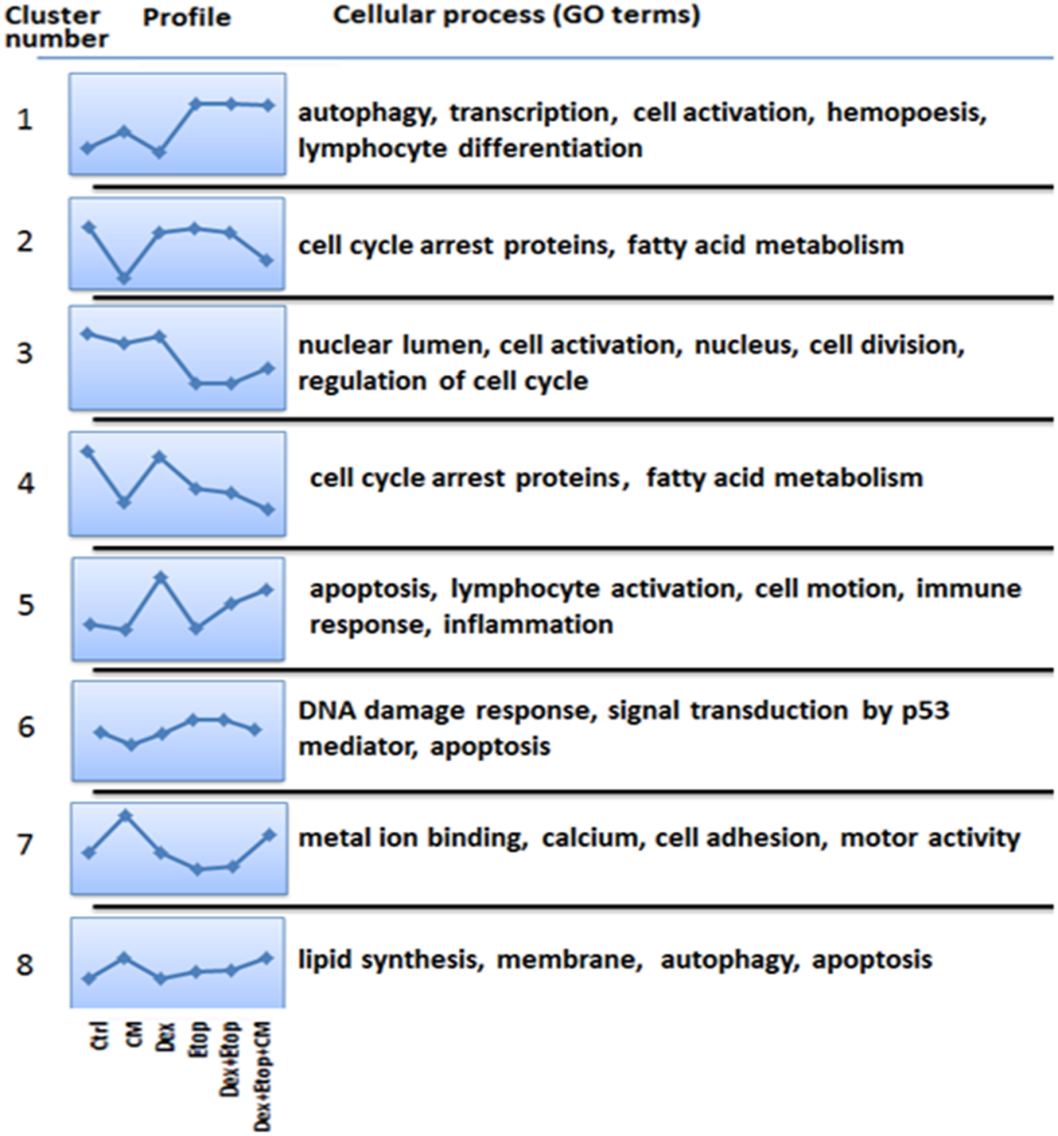
Clusters, profiles, and GO groupings of CEM-C7-14 cells. CEM-C7-14 cells were grown in the absence and presence of CM or standard RPMI media for 48h and treated with Dex (1μM) and Etop (10μM) individually or in combination for 24h. Cells were treated with vehicle (1), CM (2), Dex (3), Etop (4), Dex and Etop (5) or Dex, Etop and CM (6). Identified genes were assigned to one of eight distinct clusters using k-means clustering algorithms. On the left, the data for each cluster are represented as a profile of the z-transformed, log_2_ values for the mean of each experimental group/condition. The most significantly overrepresented GO terms are shown for the genes within each cluster.

To investigate the molecular effects of CM on ALL cellular pathways, transcriptome analysis was performed in C7 cells treated with CM, Dex and Etop in various combinations. Using fold-change as a criterion, 2632 genes showing altered expression were detected, though most were of unknown function. Therefore profile filtering was used to cluster genes into 8 categories based on expression profile similarity across treatments (Fig 1, Table 1). For each cluster, gene ontology (GO, terms indicating biological processes or cellular functions) groups were identified using the expression analysis systematic explorer to identify biologically-related gene groups based on GO term overrepresentation (Fig 1).

**Table 1:** Further clustering according to the percent of different cellular processes assigned by EASE analysis.

ClusterNo.	Lysosomes	Apoptosis	DNA Damage	Cell Cycle	Mitochondria	ER	Cytoplasmic vesicles	Phosphorylation	Inflammation	Others
**1**	**7%**	**2%**	**0%**	**2%**	**2%**	**8%**	**4%**	**0%**	**2%**	**73%**
**2**	**0%**	**6%**	**4%**	**6%**	**14%**	**0%**	**0%**	**3%**	**1%**	**66%**
**3**	**0%**	**7%**	**2%**	**5%**	**4%**	**6%**	**3%**	**7%**	**1%**	**65%**
**4**	**0%**	**4%**	**4%**	**10%**	**6%**	**2%**	**1%**	**5%**	**0%**	**68%**
**5**	**1%**	**4%**	**0%**	**0%**	**0%**	**3%**	**0%**	**0%**	**2%**	**90%**
**6**	**4%**	**12%**	**8%**	**5%**	**23%**	**0%**	**7%**	**7%**	**3%**	**31%**
**7**	**3%**	**5%**	**2%**	**6%**	**7%**	**6%**	**6%**	**7%**	**5%**	**53%**
**8**	**4%**	**6%**	**0%**	**4%**	**10%**	**31%**	**7%**	**4%**	**2%**	**47%**

Cluster 1 genes were upregulated by Etop/Dex in the presence and absence of CM and affected autophagy, haematopoiesis and lymphocyte differentiation. Cluster 3 genes were mostly downregulated by combined treatment and affected nuclear functions, cell division and cell cycle. Clusters 2/4 comprise genes involved in cell cycle control and fatty acid metabolism and were downregulated by CM. Genes upregulated in cells treated with Dex alone or in combination were associated with apoptosis, lymphocyte activation, cell motility, immune response and inflammation (Cluster 5). Cluster 7 is associated with metal binding, cell adhesion and motor activity, whereas Cluster 8 genes are involved in the regulation of lipid and membranes function, apoptosis and autophagy. Cluster 6 represents genes downregulated by CM and upregulated by DNA damage, associated with the p53 (Fig 1). Further analysis of genes that control various cellular processes suggested that cluster 6 has the highest percentage of genes involved in apoptosis, DNA damage and mitochondrial processes (Table 1). Given the importance of these processes in the response of leukemia cells to drug treatment this cluster was further analysed. Cluster 6 among others contained the Bim, Fos, Runx3 and IκB genes involved in apoptosis and these genes were downregulated in cells grown in CM. Furthermore, repression of RIPK1 and BAD gene expression was recorded in cells grown in CM. The RIPK1 ubiquitinating enzyme BIRC3 (cIAP2) gene expression was upregulated in Dex-treated C7 cells (Cluster 5). BIRC3 and RIPK1 are involved in pathways important in regulating GR such as NF-κB [46].

### Differential regulation of cell death pathways in ALL

**Fig 2:**
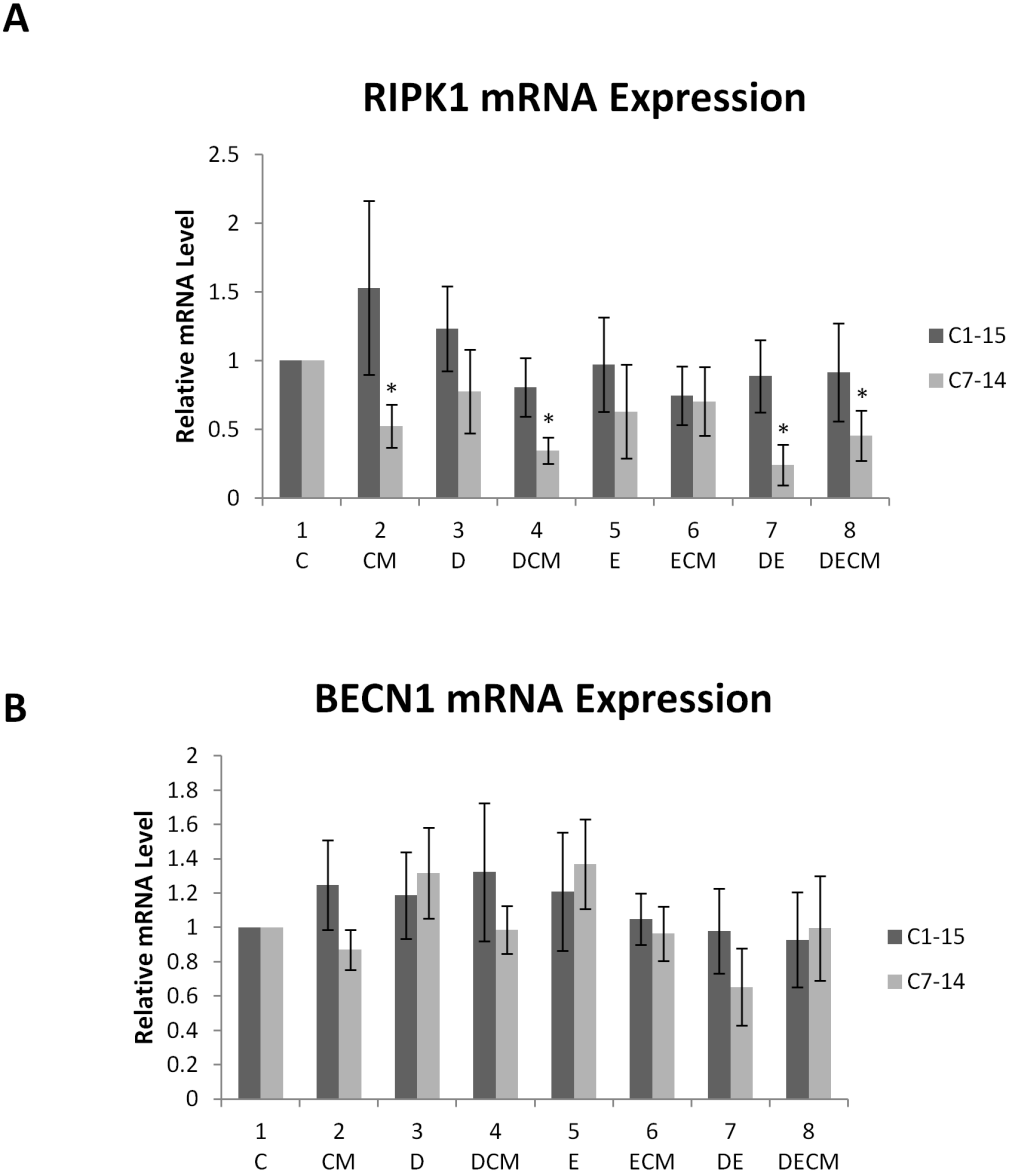
RIPK1 and BECN1 gene expression. (A) Gene expression of RIPK1 and (B) BECN1 in ALL cells treated with 1μM Dex, and 10μM Etop individually or in combination for 24h in the presence or absence of CM. The data is representative of at least three independent experiments. Error bars represent SEM. P-value of less than or equal to 0.05 is indicated by *.

**Fig 3:**
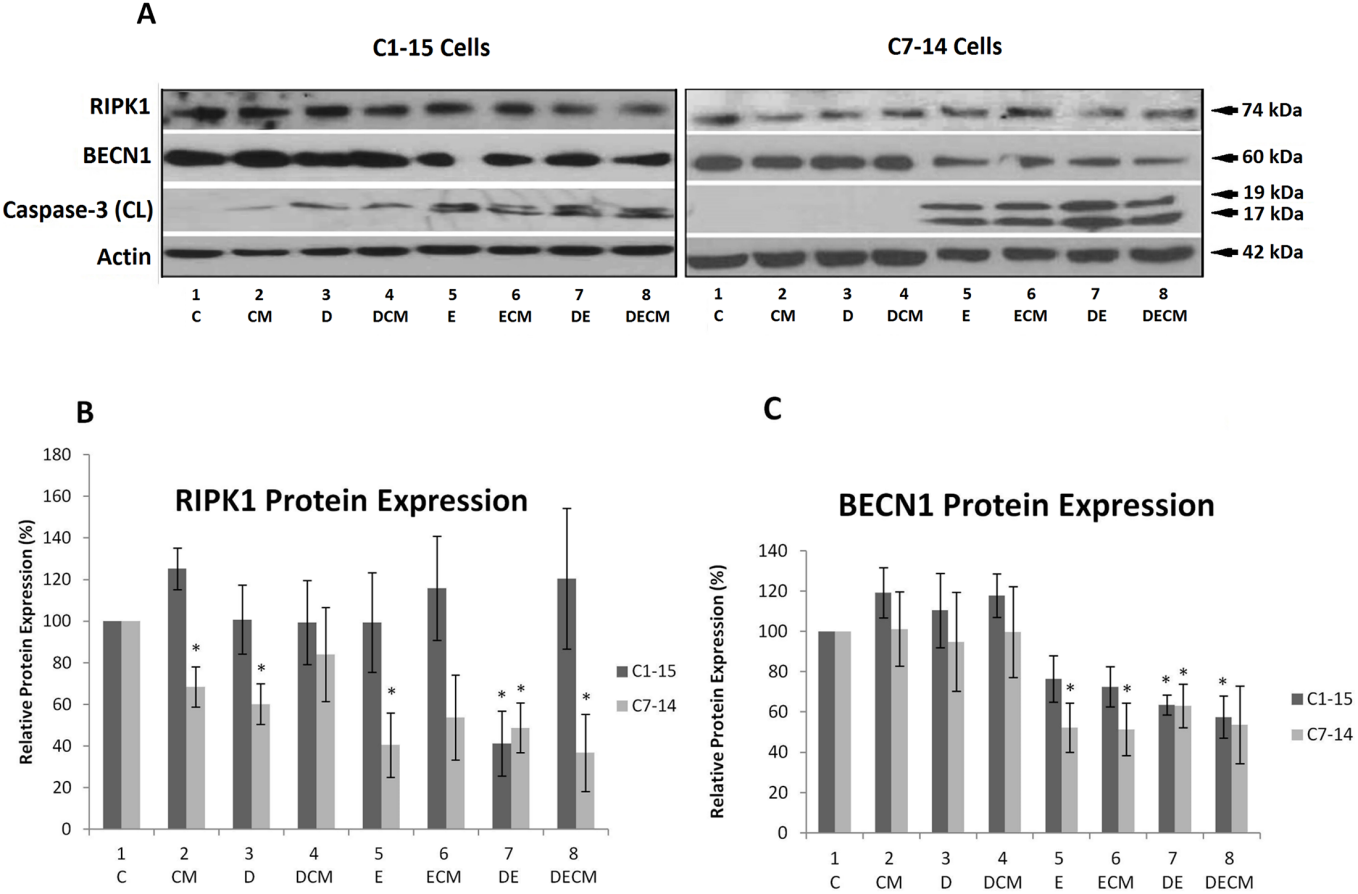
RIPK1, BECN1 and Caspase-3 protein levels in ALL cells. (A) CEM-C7-14 and CEM-C1-15 cells were grown in the absence and presence of CM or standard RPMI media for 48h and treated with Dex (1μM) and Etop (10μM) individually or in combination for 24h. Cells were lysed and analysed by SDS PAGE followed by western blot. Blots were probed with antibodies specific for RIPK1, BECN1 and cleaved caspase 3. Actin was used as a loading control. Western blots (Fig 2A and data not shown) were densitometrically scanned, normalised to actin and presented as bar charts. (B) RIPK1 protein expression; (C) BECN1 protein expression. The data is representative of at least three independent experiments. Error bars represent SEM. P-value of less than or equal to 0.05 is indicated by *. C is control; CM is conditioned media; D is Dex; DCM is Dex and CM; E is Etop; ECM is Etop and CM; DE is Dex and Etop; DECM is Dex, Etop and CM.

Microarray analysis indicated that RIPK1 was downregulated in cells grown in CM (Fig 1). RIPK1 exerts pro-survival effects when it forms the complex I together with TRADD, TRAF and cIAP proteins thereby activating NF-κB, or facilitates apoptosis/necroptosis depending on the type of complex formed with RIPK3 (complex IIa or IIb) [47, 48]. We hypothesized that CM-mediated RIPK1 downregulation could explain the development of chemoresistance. To test this we followed RIPK1 mRNA and protein levels under different treatments (Figs 2 and 3 respectively). In C1 cells, although increasing mRNA trends were observed in cells treated with CM and Dex, the change was not statistically significant (Fig 2A, compare lanes 2 and 3 with lane 1, black bars). In C7 cells CM alone or in the presence of Dex and Dex/Etoposide combination led to decrease in RIPK1 gene expression (Fig 2A compare lanes 2, 4 and 8 to lane 1). A similar effect was observed in cells treated with a combination of Dex and Etoposide (Fig 2A compare lane 7 to lane 1). BECN1 mRNA was measured to assess whether this key indicator of autophagy, was affected by these treatments (Fig 2B). BECN1 mRNA levels were altered by several treatments however, no significant changes were observed.

Alterations in the protein levels of RIPK1, BECN1 and caspase-3 were determined by western blot analysis. In C1 cells RIPK1 protein levels were downregulated in cells treated with Dex/Etop combination (Fig 3A/3B, compare dark grey bars in line 7 to other bars). In C7 cells modest and significant downregulation of RIPK1 protein levels was detected in most treatments. These observations are recapitulated to some extent in another ALL cell line MOLT4 where CM downregulated RIPK1 protein levels (Fig D in S1 File). CM affected Dex-mediated downregulation of RIPK1 (Fig 3B, compare light grey bars 4 to 3) suggesting that alterations of RIPK1 in cells grown in CM is a potential route to ALL survival or altered response to chemotherapy.

Protein levels of apoptosis (caspase-3) and autophagy (BECN1) markers were followed to determine the effect of CM on cell death and survival in ALL upon Dex/Etop treatment [49] (Fig 3). Analysis of these markers (Fig 3C) indicated that BECN1 protein levels were not significantly affected in either cell line grown in CM with or without Dex (Fig 3A and 3C, lanes 1–4). Repression of BECN1 was observed in C1 cells treated with Dex/Etop combination (Fig 3 compare dark grey bar in lane 7 to other bars). Reduction of BECN1 protein levels was observed in Etop, Etop/CM and Dex/Etop-treated C7 cells, whereas Dex/Etop/CM showed no statistically significant loss (Fig 3C compare light grey bar 8 to bars 5, 6 and 7). These results suggest that CM reverses the inhibitory effect of Etoposide on BECN1 protein levels in C7 cells. However, C1 cells were insensitive to CM as the downregulation of BECN1 observed in these cells treated with Dex/Etop was not reversed by CM (Fig 3, compare dark grey bars of lanes 7 and 8), suggesting that CM exerts cell-specific effects on BECN1.

No major changes in cleaved caspase-3 were observed in C1 cells apart from an elevation exhibited in cells treated with Etoposide (Fig 3A). Cleaved caspase-3 was detected at very low levels in C7 cells treated with all combinations except those containing Etoposide (Fig 3A and data not shown). Decreased protein levels of cleaved caspase-3 were recorded in C7 cells grown in CM and treated with combination of Dex and Etop compared to cells grown in normal media and treated with combination of Dex and Etop, suggesting that CM potentially exerts pro-survival effects (Fig 3A compare lanes 7 and 8). Taken together, these results indicated that CM exerts inhibitory effects on the cell death mediators RIPK1 and caspase-3 and possibly stimulates pro-survival autophagy.

### The microenvironment alters GR phosphorylation

**Fig 4:**
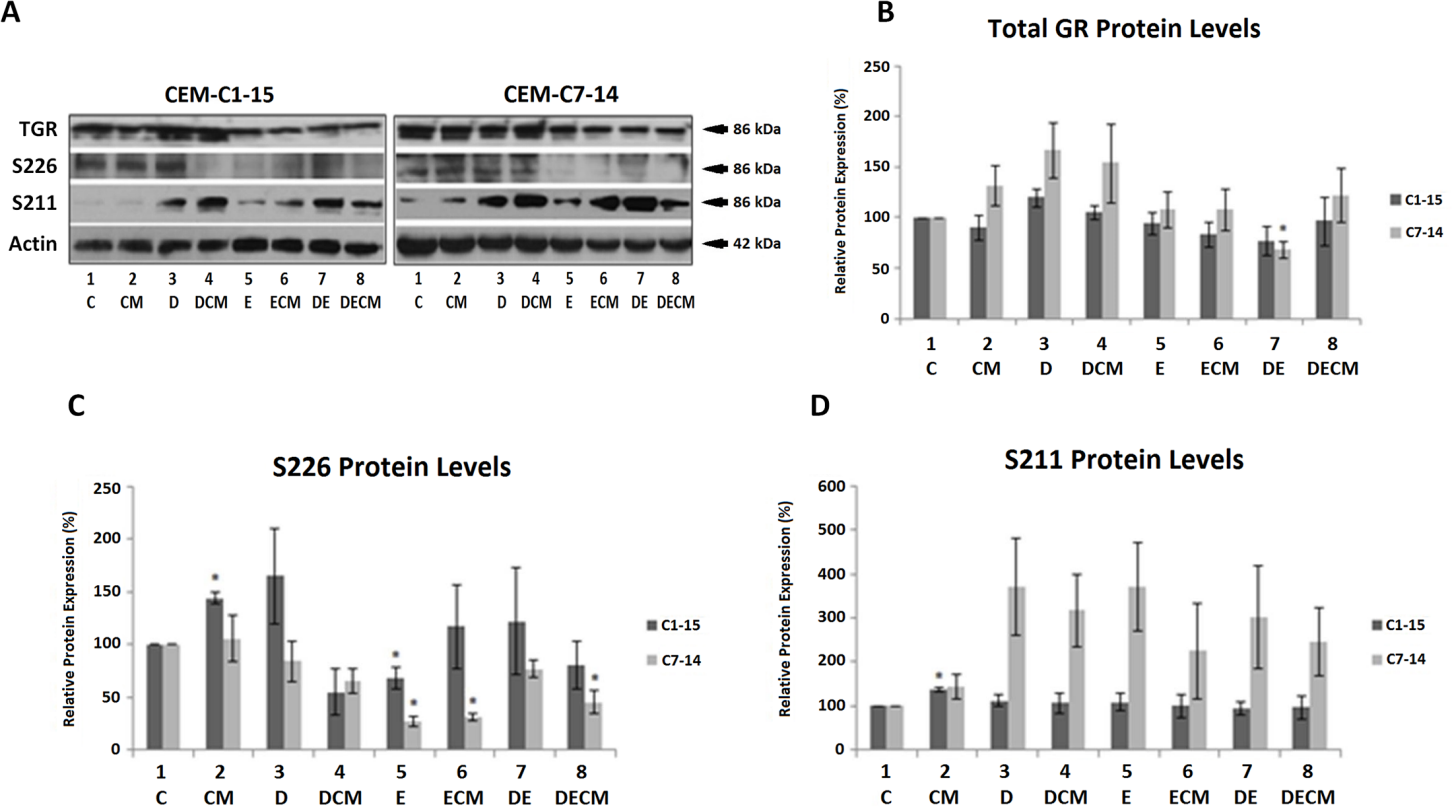
Dex, Etop and the microenvironment affect GR phosphorylation in ALL. (A) CEM-C1-15 and CEM-C7-14 cells were cultured in CM or standard RPMI media for 48h and treated with Dex (1μM) and Etop (10μM) individually or in combination for 24h. Treatments were as in Fig 2. Cells were lysed and protein extracts were subjected to western blot analysis. Total and phosphorylated GR was detected using specific antibodies against these proteins. Actin was used as loading control. Western blots (Fig 4A and data not shown) were densitometrically scanned, normalised to actin and presented as bar charts. For phosphorylation experiments, phosphorylation blot data was scanned and normalised to the actin-normalised total GR. (B) Total GR protein expression. (C) S226-phosphorylated GR protein expression. (D) S211-phosphorylated GR protein expression. The data is representative of at least three independent experiments. Error bars represent SEM. P-value of less than or equal to 0.05 is indicated by *.

To investigate whether CM affects GR phosphorylation, thereby mediating sensitivity or resistance in ALL cells [27, 28, 46] total GR protein levels and its phosphoisoforms S211 and S226 were monitored in ALL cells following Dex and Etop treatment (Fig 4, data not shown).

Although some increase in total GR levels in the presence of Dex and decrease in the presence of Etop were observed, these changes were marginal and mostly insignificant, except when C7 cells were treated with Dex and Etoposide (Fig 4A and 4B, lane 7 light grey bars). S226-phosphorylated GR increased in CM and Dex-treated C1 cells, whereas Dex/CM combined treatment downregulated S226 phosphorylation (Fig 4A and 4C, compare lanes 1–4, left panel). S226 phosphorylation was mostly downregulated in the presence of Etoposide individually or in combination with other treatments in C1 cells, with significant effects recorded in Etoposide treated cells (Fig 4A and 4C, compare lanes 5, 6, 7 and 8 to lane 1). However, effects of Dex and CM on S226 phosphorylation were not recapitulated in C7 cells as Dex, CM and combination Dex/CM exerted minor effects (Fig 4A and 4C, compare lanes 1–4). Etoposide alone or with co-treatments downregulated S226 phosphorylation in C7 cells (Fig 4A and 4C, compare lanes 5–8 to lane 1).

The efficiency of GR phosphorylation at S211 was higher in C7 than in C1 cells under most conditions. Increase in S211-phosphorylated GR was observed in C1 cells grown in CM, whereas no further changes were observed in C1 cells exposed to other treatments (Fig 4A and 4D, dark grey bars). Increased S211-phosphorylated GR was evident in Dex and Etoposide treated C7 cells (Fig 4A and 4D, compare lanes 3–8 to 1, light grey bars). In C7 cells, when CM was added to cells treated with Dex and Etoposide, trends of decreased levels of phosphorylation were detected upon quantification of multiple blots (Fig 4D, compare lanes 3, 5, 7 with lanes 4, 6, 8).

### Recruitment of GR on RIPK1 and BECN1 promoters

**Fig 5:**
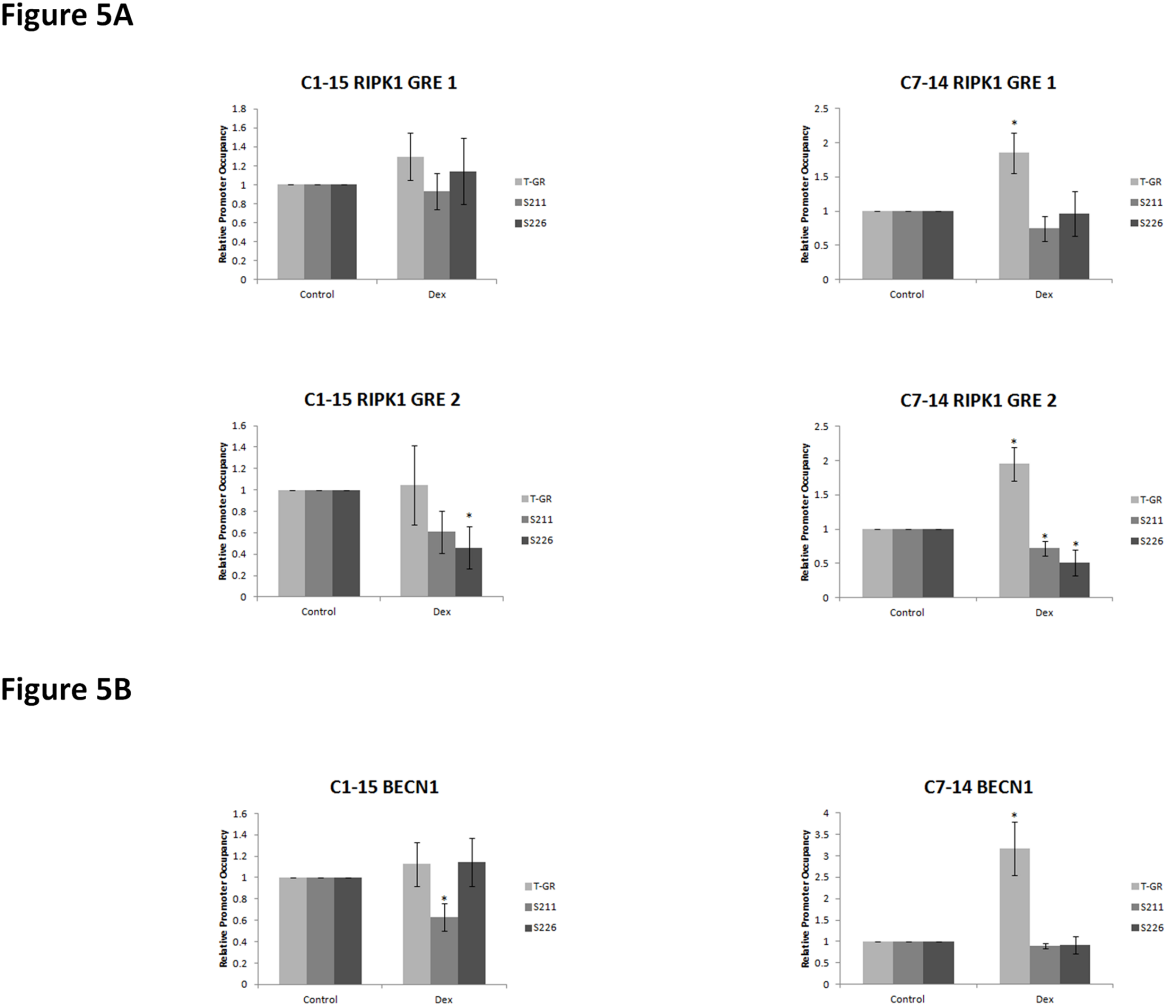
Differential recruitment of GR and its phosphoisoforms on the RIPK1 and BECN1 promoters. Chromatin immuniprecipitation (ChIP) analysis was carried out in CEM-C1-15 and CEM-C7-14 cells treated with 1μM Dex for 24hrs. We identified potential GR binding sites on the (A) RIPK1 and (B) BECN1 promoters and analysed total GR, and GR phosphorylated on S211 and S226 on a subset of sites. The data is representative of at least three independent experiments. Error bars represent SEM. P-value of less than or equal to 0.05 is indicated by *.

Recruitment of GR and its S211/S226 phosphoisoforms to the RIPK1 and BECN1 promoters was analysed using ChIP assay. Two putative GREs were identified in the RIPK1 promoter using the Qiagen Champion ChiP Transcription Factor Search Portal. GR recruitment was detected on both GREs of the RIPK1 promoter in Dex-treated C7 cells (Fig 5A). Surprisingly, GR phosphoisoforms recruitment to the GRE2 of the RIPK1 promoter decreased in the presence of hormone (Fig 5A).

Sequence analysis of BECN1 revealed the existence of numerous potential GREs in its promoter region (thirteen were identified by Qiagen Champion ChIP). No significant GR recruitment to BECN1 GREs was evident in the Dex-treated compared to untreated C1 cells whereas S211-phosphorylated GR was recruited slightly less compared to untreated cells (Fig 5B, left panel). Increased recruitment of total GR but not GR phosphorylated at S211 was evident in Dex-treated C7 cells (Fig 5B, right panel).

### BIRC3 gene expression

**Fig 6:**
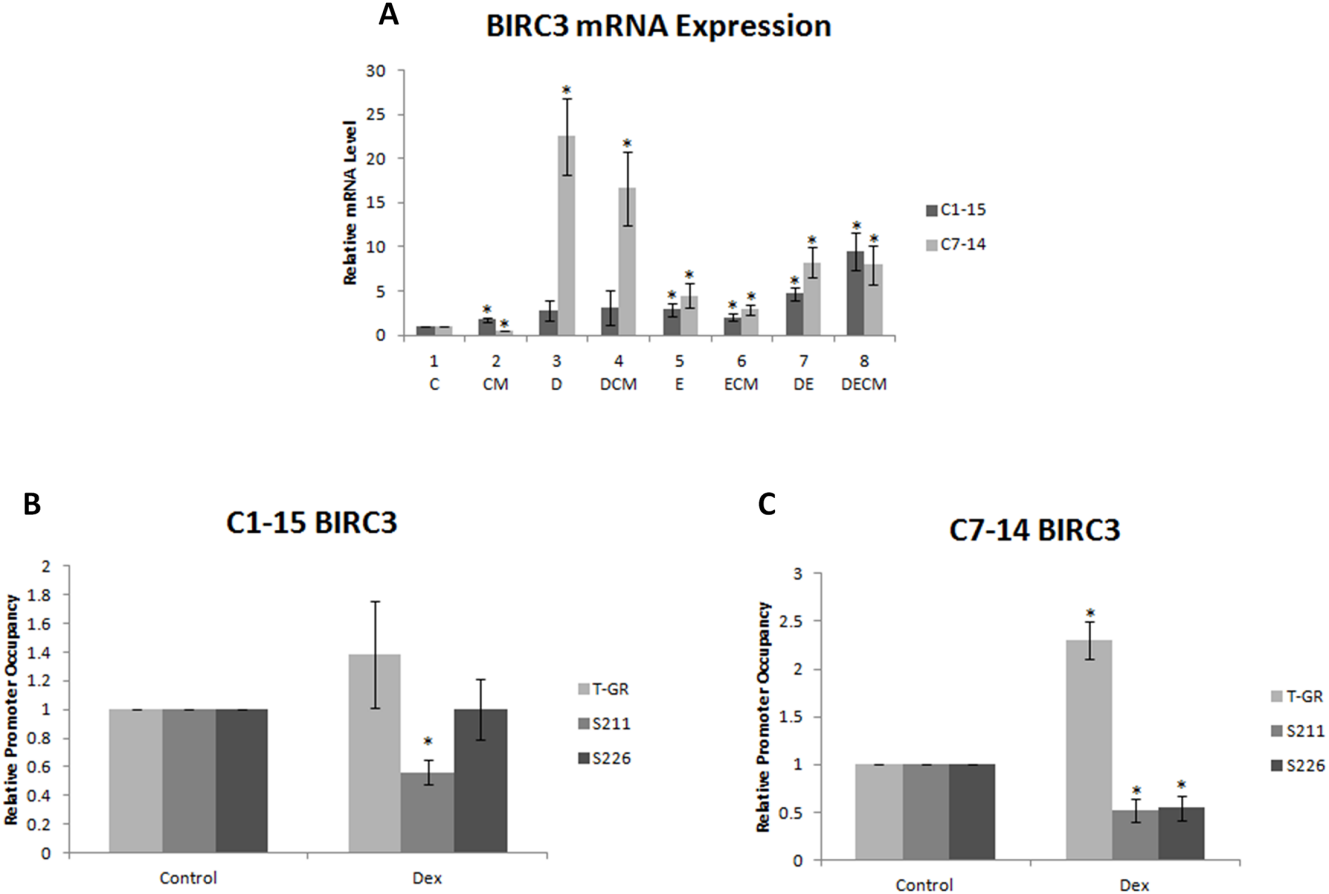
Control of BIRC3 gene expression by GR in ALL. (A) mRNA expression of BIRC3 in ALL cells treated with Dex, Etop and CM. (B) ChIP analysis was carried out in CEM-C1-15 and (C) CEM-C7-14 cells treated with 1μM Dex for 24hrs. Occupancy on one of the GREs by total GR, and GR phosphorylated on S211 and S226 was analysed. The data is representative of at least three independent experiments. Error bars represent SEM. P-value of less than or equal to 0.05 is indicated by *.

Ubiquitination/deubiquitination cycles of RIPK1 define the multiprotein complexes formed by this protein, determining whether cells survive or undergo necroptosis/apoptosis. Formation of the pro -survival complex I requires that RIPK1 is ubiquitinated by BIRC3, leading to NF-κB activation and cell survival [47]. Alternatively, RIPK1 deubiquitination results in the formation of the complex IIa inducing apoptosis [48]. When caspase-8 activity is blocked, and caspase-8-mediated cleavage of RIPK1 does not take place, RIPK1 binds to RIPK3 (complex IIb) and necrotic signalling is triggered [48]. BIRC3 gene expression was followed in Dex-treated C1 and C7 cells to validate BIRC3 upregulation observed in microarray experiment in Dex-treated C7 cells and gain insight in molecular aspects of RIPK1 signalling. Upregulation of BIRC3 gene expression was observed in C1 cells grown in CM and more prominent upregulation of the expression of this gene was observed in the presence of Etoposide alone or in combination with Dex and CM (Fig 6A, compare dark grey bars, lane 2 to 1 and lanes 5–8 to 1). CM alone led to downregulation of BIRC3 mRNA in C7 cells (Fig 6A, compare lane 2 to lane 1 light grey bars). BIRC3 was substantially upregulated in C7 cells treated with Dex or CM (Fig 6A, light grey bars 3 and 4). Dex and Etop with/without CM upregulated BIRC3 gene expression but to a lesser extent than cells treated with Dex only (Fig 6A, compare lanes 7/8 with lane 1/3, light grey bars).

No change in total GR and S226-phosphorylated GR recruitment on BIRC3 promoter was observed in Dex-treated C1 cells whereas decreased recruitment of the S211-phosphorylated GR in these cells was evident (Fig 6B). In contrast, substantial Dex-dependent recruitment of total GR and modest decrease in phosphorylated GR was recorded in C7 cells (Fig 6C).

### Effects of Dex, Etop and the microenvironment on leukaemia cell fate

**Fig 7:**
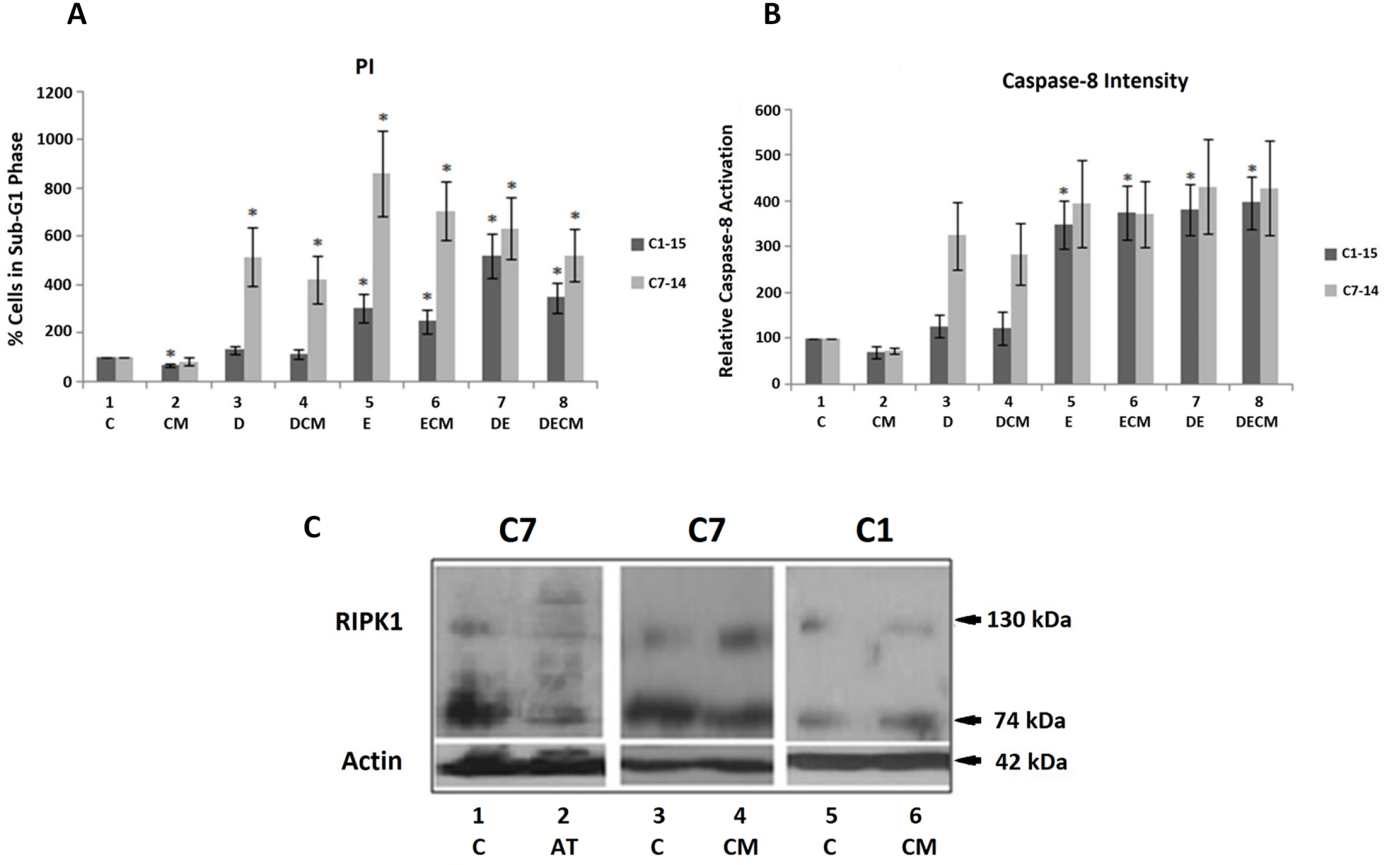
Microenvironment and chemotherapy effects on ALL cell fate. (A) Cells were treated with CM (48 hours), 1μM Dex (36 hours) and 10μM Etop (24 hours) in varying combinations. Percentage of cell death was obtained using FACS analysis of PI-stained CEM-C1-15 and CEM-C7-14 cells treated as above. SubG1 phase is shown. (B) Caspase-8 activity was measured in ALL CEM-C7-14 and CEM-C1-15 cells treated as indicated. CEM-C1-15 cells are represented by dark grey bars, CEM-C7-14 by light grey bars. (C) RIPK1 high molecular weight forms were analysed in cells treated with CM and BIRC3 inhibitor AT406 (10μM for 48 hours). Western blot analysis was carried out as described above. The data is representative of at least three independent experiments. Error bars represent SEM. P-value of less than or equal to 0.05 is indicated by *.

Effects of different treatments on ALL cell cycle progression were investigated using FACS analysis and PI staining. CEM-C7-14 cells exhibited higher sensitivity to glucocorticoids compared to CEM-C1-15 cells as estimated by the number of cells accumulating in the SubG1 phase (Fig 7A). Combination of Dex with CM, or Dex/Etop with CM resulted in reduction of the sensitivity of CEM-C7-14 cells to treatment (Fig 7A, compare grey bars 4/8 to grey bars 3/7 respectively). Similar effect of CM was observed in CEM-C1-15 cells but to a lesser extent than that detected in the CEM-C7-14 cells (Fig 7A, compare black bars 4, 6 and 8 to black bars 3, 5 and 7 respectively) indicating the potential role of CM in conferring resistance to leukaemia cells in a cell and type of treatment-dependent manner.

The interplay between caspase-8 and BIRC3 is crucial in determining cellular pathways leading to survival or apoptosis/necroptosis [17, 18]. In order to investigate which of these pathways is predominant in C1 and C7 cells under various conditions, caspase-8 activity was determined in these cells (Fig 7B). In C1 cells, caspase-8 was upregulated in the presence of Etoposide alone or in combination of Dex with CM (Fig 7B, compare dark grey bars lanes 5–8 to 1). Increasing trend of caspase-8 intensity was observed in C7 cells upon Dex or Etoposide treatment individually or in combination (Fig 7B, compare light grey bars lanes 3–8 to lane 1).

Given that BIRC3 serves as ubiquitin ligase for RIPK1, AT406 BIRC3 inhibitor was used to assess the RIPK1 protein levels in C7 cells (Fig 7C). Longer exposure of the blots revealed several bands of higher molecular weight that interacted with RIPK1 antibody. Band of about 130kDa increased in intensity in C7 cells incubated in CM when compared to C1 cells (Fig 7C, compare lanes 3 and 4 to 5 and 6). Intensity of high molecular weight bands was modified in cells treated with the BIRC3 inhibitor AT406 (Fig 7C, compare lane 1 to lane 2) implying that these bands possibly represent ubiquitinated RIPK1 forms. Taken together these data suggest that conditioned medium diminishes the effects of Dex and Etop on ALL cells and that this process is at least in part executed through alterations of RIPK1 ubiquitination.

## Discussion

The effects of the microenvironment on ALL cells were investigated by microarray analysis of these cells grown in CM and treated with Dex and/or Etop. CM inhibited RIPK1 and caspase-8/3, and interfered with chemotherapy-induced downregulation of BECN1. GCs induced the RIPK1 ubiquitinating enzyme BIRC3 only in GC-sensitive cells. CM altered GR phosphorylation state providing potential link between microenvironment and drug response. GR was recruited preferentially on RIPK1, BECN1 and BIRC3 promoters in C7 cells with lower amount of the phosphorylated GR occupancy in the presence of hormone. CM showed tendency to increase cell survival and increased high molecular weight RIPK1 forms that were sensitive to AT406 (BIRC3 inhibitor).

Although genome-wide analyses of differentially expressed GC- regulated genes have been reported, limited information exists regarding the effects of the microenvironment on chemoresistance [13, 38, 50–52]. Transcriptome analysis of GC-sensitive cells treated with Dex, Etop and CM identified genes exhibiting altered expression [53, 54]. BCL2-associated agonist of cell death (BAD) and the nuclear factor of kappa light polypeptide gene enhancer in B-cells inhibitor beta (I-κB) were repressed by CM. I-κB is an NK-κB inhibitor therefore inhibition of its activity by CM could lead to survival/resistance. RIPK1, related to different forms of cell death [55] as well as to pathways important for the regulation of GR function such as the MAPK and NF-κB, was repressed in cells grown in CM. Furthermore, transcriptome analysis indicated that the RIPK1 ubiquitinating enzyme BIRC3 was upregulated in cells treated with Dex (Fig 1). CM repressed RIPK1 levels in C7 cells to a greater extent than in C1 (Figs 2 and 3), suggesting that this might be a path to chemoresistance given the role of RIPK1 in necroptosis [56], NF-κB [57] and GR [16] signalling.

The large number of putative GREs within the BECN1 promoter suggests that crosstalk between BECN1 and GR might exist and that BECN1 may be involved in GC-induced cell death [58]. Downregulation of BECN1 protein levels by Dex and Etop was abrogated in the presence of CM in C7 cells (Fig 3 compare lanes 7 and 8 to lane 1), suggesting that CM-dependent BECN1 upregulation leads to induction of pro-survival autophagy and protection from chemotherapy.

Downregulation of cleaved caspase-3 was observed in C7 cells grown in CM and treated with both Dex and Etop (Fig 3). This could indicate a pro-survival pathway induced by CM since the phosphatidylinositol-4-phosphate 3-kinase (PIK3C2B) that blocks caspase-3 was upregulated by CM [59–61]. In summary, CM downregulates RIPK1 and caspase-3, mediators of necroptosis and apoptosis respectively, and increases the pro-survival BECN1 protein levels in C7, but not in C1 cells, thereby promoting survival of the sensitive cells.

CM displayed cell and site specific effects on GR phosphorylation. It is possible these effects are mediated through modulation of kinases known to affect GR. JNK-mediated phosphorylation of GR at S226 plays an important role in reversing GC-sensitivity in resistant T-cells whereas inhibition of GR phosphorylation at S226 potentiates its transcriptional activity [10, 20, 23, 27, 62, 63]. Higher ratio of S211/S226 GR phosphorylation has been shown in C7 compared to C1 cells, coinciding with altered NOXA/Mcl-1 gene expression in sensitive cells, implying that site—specific GR phosphorylation determines GC sensitivity [10]. The hypothesis of altered gene expression by differentially-phosphorylated GR thereby leading to the induction of pro—survival pathways was investigated employing ChIP to determine the recruitment of GR and its phosphoisoforms to the RIPK1, BECN1 and BIRC3 promoters. Increased recruitment of total GR occurred at all three promoters when antibody against total GR protein was used, however, there was decreased recruitment of GR phosphoisoforms in hormone treated C7 cells (Figs 4 and 5). These data suggested that GR may affect gene expression of these genes however no statistically significant effects were observed when mRNA levels were determined for RIPK1 and BECN1 (Fig 2). This is potentially due to complex control of BECN1 given the existence of 13 putative GREs in its promoter and numerous levels of crosstalk between GR and NF-κB that is a RIPK1 downstream target. No recruitment of GR to the promoters of these genes was observed in C1 cells.

TNF-alpha mediated apoptosis involves the formation of complex I which contains ubiquitinated RIPK1, TRADD, TRAF2 and BIRC3 [64–66]. Complex I formation activates NF-κB leading to cell survival. Increased recruitment of GR on BIRC3 promoter coinciding with marked induction of this gene expression in Dex-treated C7 cells (Fig 6) raises the possibility that ALL resistance to drug treatment could be reversed by applying combinatorial treatments of Dex with BIRC3 inhibitors. Caspase-8, induced by Dex in C7 cells only, is also involved in this pathway. However, Etop co-treatment with Dex changes BIRC3 induction and brings it to similar levels in both C1 and C7 cells, highlighting the molecular basis for the beneficial effect of combination therapy in the clinic.

Activation of caspase-8 by Dex could induce cleavage of RIPK1 and RIPK3 and formation of the complex II which contains RIPK1, RIPK3, caspase-8 and FADD and leads to apoptosis. Additionally Dex upregulates cFLIP in hepatocytes therefore cFLIP upregulation in C7 cells may lead to caspase-8 inhibition and stimulation of necroptosis [67, 68]. Necrosome formation via stimulation of TRAIL/TNF activates RIPK3 which interacts with enzymes regulating glycolytic flux and glutaminolysis [68]. Reactive oxygen species ultimately form, whilst redox status is important in determining sensitivity to microenvironment and chemotherapy [35, 67–69]. RIPK1 substrates are yet to be identified as are factors necessary for cell death induction through necroptosis or apoptosis [55].

Although the CM constituents are not fully known, they may include membrane receptor activators, miRNAs and exosomes [35]. CM may affect RIPK1 potentially leading to altered GR and other proteins phosphorylation levels. Alternatively, miRNA-mediated modulation of kinases targeting GR could explain its differential phosphorylation patterns in the sensitive versus resistant ALL cells. It is evident that RIPK1 is a target of direct (promoter binding) and indirect (BIRC3 upregulation) GR activity. It is also possible that CM increases RIPK1 ubiquitination thus promoting survival and potentially affecting NFκB signalling. Our data suggest that the microenvironment affects components of the autophagy, apoptosis and necroptosis pathways favouring survival and chemoresistance. Effective treatment of ALL may be achieved through therapies targeting components of the microenvironment.

## Conclusions

This study provided evidence to suggest that several components of apoptosis, autophagy and necroptosis are regulated by the tumour microenvironment in a manner dependent on the post-translational modification profile of the GR. These effects of the microenvironment correlate with the outcome of the response to particular chemotherapeutic treatments. Further studies are needed to identify the details of the causative relationships between tumour microenvironment and response to chemotherapy in order for these findings to be utilised in the clinic.

## Supporting information

S1 File This is the S1 File containing the Table A, the Table B and Fig A, Fig B, Fig C and Fig D. (DOCX)
