# Supplementary material for: Differential regulation of cell death pathways by the microenvironment correlates with chemoresistance and survival in leukaemia
Source: PLoS One. 2017 Jun 5;12(6):e0178606. doi: 10.1371/journal.pone.0178606 (PMC5459454; doi:10.1371/journal.pone.0178606)
Supplement: S1 File — (DOCX) [file pone.0178606.s001.docx]

**Supporting Information S1**

**Table A. The table shows the primers used for qRT-PCR to determine the mRNA levels of the indicated genes.**

| **Name of Gene** | **Primer** | **Sequence** |
| --- | --- | --- |
| BECN1 | Forward | TTG GCA CAA TCA ATA ACT TCA GGC |
|  | Reverse | CCG TAA GGA ACA AGT CGG TAT CTC |
| BIRC3 | Forward | ACT TGA ACA GCT GCT ATC CAC ATC |
|  | Reverse | GTT GCT AGG ATT TTT CTC TGA ACT GTC |
| RIPK1 | Forward | TGG AAA AGG CGT GAT ACA CA |
|  | Reverse | GAC TTC TCT GTG GGC TTT GC |
| RPL19 | Forward | ATG TAT CAC AGC CTG TAC CTG |
|  | Reverse | TTC TTG GTC TCT TCC TCC TTG |

**Table B. The table shows the primers used to amplify the putative GRE regions within the indicated genes in the ChIP experiments.**

| **Name of Gene** | **Primer** | **Sequence** |
| --- | --- | --- |
| BECN1 GRE 1 | Forward | ACT CCT GAC CTT GTG ATC CG |
|  | Reverse | AGA ATC GCT TGA ACC TGG GA |
| BIRC3 GRE | Forward | AAG ATG TGT TAG CCA GTC CTG TT |
|  | Reverse | CCC AAT TTT TCT CCA ATT AGT CA |
| RIPK1 GRE 1 | Forward | CTC CGC AGC TCC CAG C |
|  | Reverse | TGG GTA AGA GTG CTC GGA TT |
| RIPK1 GRE 2 | Forward | AGT CTT GCT CTG TCA CCC A |
|  | Reverse | GGT GAA GCC CTG TCT CTA CT |

A

B

C


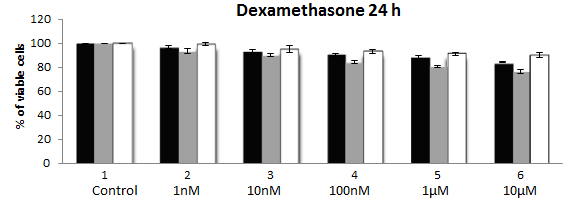

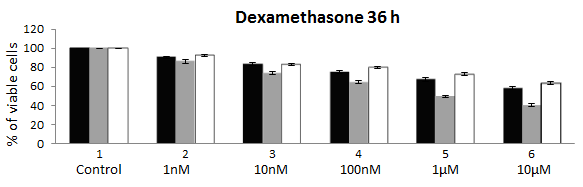

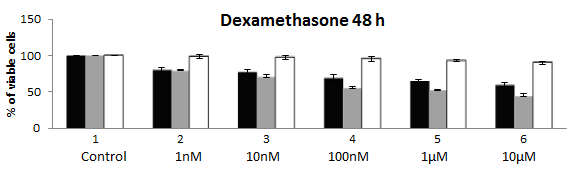

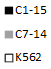


**Fig A. Determination of the optimal dose and duration of glucocorticoid treatment of leukaemia cells.**

(A) CEM-C1-15 (black bars), CEM-C7-14 (grey bars) and K562 cells (white bars) were treated with different concentrations of Dex for 24h (B) 36h and (C) 48h. Cell viability was determined using trypan blue exclusion and a haemocytometer. The data is representative of 3 independent experiments. Error bars represent standard deviation.

A

B

C


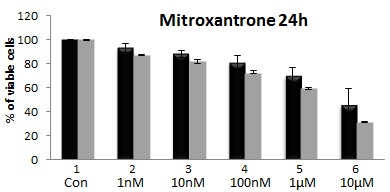

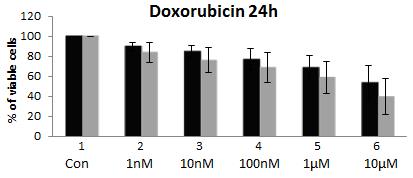

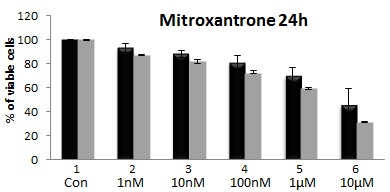

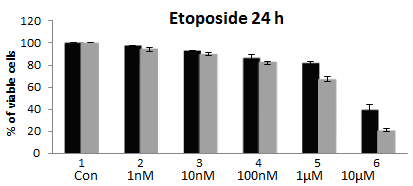


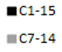


**Fig B. Determination of the optimal type, dose and duration of anthracycline treatment of leukaemia cells.**

(A) CEM-C1-15 (black bars) and CEM-C7-14 cells (grey bars) were treated with different concentrations of Etoposide (B) Mitroxantrone or (C) Doxorubicin for 24h. Cell viability was determined using trypan blue exclusion and a haemocytometer. The data is representative of 3 independent experiments. Error bars represent standard deviation.

**Fig C. The effect of bone marrow microenvironment on ALL cells.**

The effect of combined treatment of CM (48 hours), Dexamethasone (36h) and Etoposide (24h) on CEM-C1-15 (black bars) and CEM-C7-14 cells (grey bars) was determined using trypan blue assay and a haemocytometer. The data is representative of 3 independent experiments. Error bars represent standard deviation.


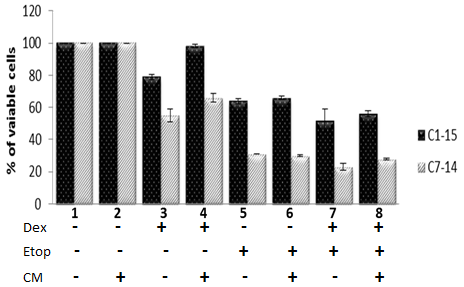


A

**
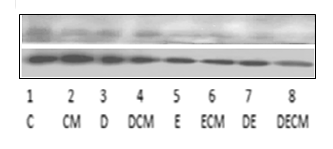
**

RIPK1

Actin

B

**
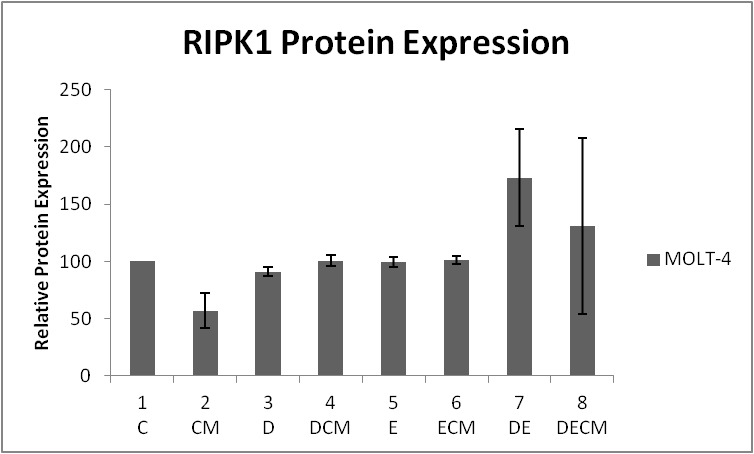
**

**Fig D. RIPK1 protein levels in MOLT4 cells.**

(A) MOLT4 cells were grown in the absence and presence of CM or standard RPMI media for 48h and treated with Dexamethasone (1μM) and Etoposide (10μM) individually or in combination for 24h. Cells were lysed and analysed by SDS-PAGE followed by western blot. Blots were probed with antibodies specific for RIPK1. Actin was used as a loading control. (B) Western blots (S1 Fig 4A and data not shown) were densitometrically scanned and analysed using Image J. Data is the average of two experiments. Error bars represent standard error of means.
